# 3,4-Dicaffeoylquinic Acid, a Major Constituent of Brazilian Propolis, Increases TRAIL Expression and Extends the Lifetimes of Mice Infected with the Influenza A Virus

**DOI:** 10.1155/2012/946867

**Published:** 2011-08-25

**Authors:** Tomoaki Takemura, Tomohiko Urushisaki, Mayuko Fukuoka, Junji Hosokawa-Muto, Taketoshi Hata, Yumiko Okuda, Sachie Hori, Shigemi Tazawa, Yoko Araki, Kazuo Kuwata

**Affiliations:** ^1^Nagaragawa Research Center, API Co., Ltd., 692-3 Nagara, Gifu 502-0071, Japan; ^2^United Graduate School of Drug Discovery and Medical Information Sciences, Gifu University, 1-1 Yanagido, Gifu 501-1193, Japan; ^3^CREST, Japan Science and Technology Agency, 4-1-8 Honcho, Kawaguchi, Saitama 332-0012, Japan; ^4^Center for Emerging Infectious Diseases, Gifu University, 1-1 Yanagido, Gifu 501-1194, Japan; ^5^First Department of Forensic Science, National Research Institute of Police Science, 6-3-1 Kashiwanoha, Kashiwa, Chiba 277-0882, Japan

## Abstract

Brazilian green propolis water extract (PWE) and its chemical components, caffeoylquinic acids, such as 3,4-dicaffeoylquinic acid (3,4-diCQA), act against the influenza A virus (IAV) without influencing the viral components. Here, we evaluated the anti-IAV activities of these compounds *in vivo*. PWE or PEE (Brazilian green propolis ethanol extract) at a dose of 200 mg/kg was orally administered to Balb/c mice that had been inoculated with IAV strain A/WSN/33. The lifetimes of the PWE-treated mice were significantly extended compared to the untreated mice. Moreover, oral administration of 3,4-diCQA, a constituent of PWE, at a dose of 50 mg/kg had a stronger effect than PWE itself. We found that the amount of tumor necrosis factor-related apoptosis-inducing ligand (TRAIL) mRNA in the mice that were administered 3,4-diCQA was significantly increased compared to the control group, while H1N1 hemagglutinin (HA) mRNA was slightly decreased. These data indicate that PWE, PEE or 3,4-diCQA possesses a novel and unique mechanism of anti-influenza viral activity, that is, enhancing viral clearance by increasing TRAIL.

## 1. Introduction

Influenza is a common infectious disease, and various anti-influenza drugs are already on the market. For example, M2 ion channel inhibitors (amantadine and rimantadine) and neuraminidase inhibitors (zanamivir, oseltamivir, etc.) have been used to treat influenza viral infections in many areas, including the United States, Europe, and Asia [1–3]. Although oseltamivir is one of the most popular anti-influenza drugs, many resistant strains have already been discovered [4, 5]. Fortunately, the resistant strains that have been found to date are not yet pandemic. However, we need to carefully monitor the emergence and prevalence of new strains with pandemic characteristics, such as the H1N1 pandemic with drug resistance, which may occur because of the high incidence of mutations [6] and genomic rearrangements [7]. To avoid the worst, the discovery of novel anti-influenza agents that do not carry the risk of creating resistant strains would be urgently required.

Propolis, a resinous substance collected by honeybees from various plant sources, is used by the bees to protect the nest entrance against intruders and bacteria. Since ancient times, it has been used as a traditional folk medicine in many countries. Recent studies have demonstrated that propolis has a wide range of pharmacological properties, including antibacterial [8–10], antioxidant [11], anti-inflammatory [12], and antitumor activities [13]. The activity and constituents of propolis vary with its geographical origin [14]. The differences in its constituents arise from differences in the plants from which it is sourced [15].

The antiviral activity of propolis has been reported and includes anti-BBMV [16], anti-HSV [17–21], antipoliovirus [22], anti-IBDV [10], anti-reovirus [10], antipotato viruses [23], and anti-HIV [24] activities [25, 26]. The anti-influenza activity of propolis has been also described in several reports [27–30], but the details of its activity were different. The reason for this may be differences in the plant origin of the propolis [14].

Recently, we have demonstrated that Brazilian green propolis water extract (PWE) and its components, particularly 3,4-dicaffeoylquinic acid (3,4-diCQA), have anti-IAV activity [31]. Moreover, we found that a central mechanism of these compounds is the enhancement of cellular viability via unknown cellular processes rather than activity against the viral components [31]. 

Here, we examined the *in vivo* effects of propolis extracts on the lifetimes of mice infected with the IAV. To obtain insights into the mechanism of the antiviral effects of PWE and PEE, we examined tumor necrosis factor-related apoptosis-inducing ligand (TRAIL) and IAV hemagglutinin (HA) gene expression in the lungs, because it has been reported that the ethanol extract of Polish propolis has anti-tumor activity via TRAIL enhancement [32].

## 2. Methods

### 2.1. Reagents and Compounds

Oseltamivir phosphate (Tamiflu) was purchased from the Chugai Pharmaceutical Co., Ltd. (Tokyo, Japan). Chlorogenic acid was purchased from the Tokyo Chemical Industry Co., Ltd. (Tokyo, Japan). All media and reagents for cell culture were purchased from Invitrogen (Carlsbad, CA, USA), Sigma (St. Louis, MO, USA), and Wako Pure Chemicals (Osaka, Japan). Water extracts of Brazilian green propolis (Minas Gerais State, Brazil) originating from *Baccharis dracunculifolia* [33] were obtained from the API Co., Ltd. (Gifu, Japan). 3,4-diCQA (99% or 82% purity) was purified from 25% ethanol-extracted Brazilian green propolis via column chromatography and HPLC (details not shown).

### 2.2. Mice

Female BALB/c mice (5 weeks old) were obtained from Japan SLC, Inc. (Hamamatsu, Japan) and housed at room temperature (maintained at 23 ± 3°C) with a relative humidity range of 32–64% and a regular 12 hr light/dark cycle. The mice were fed a CE-2 rodent diet from CLEA Japan Inc. (Tokyo, Japan) and allowed free access to water.

### 2.3. Viruses

The influenza virus wild-type strain A/WSN/33 (H1N1), generated from cloned cDNAs using plasmid-based reverse genetics [34], was kindly supplied by Dr. Yoshihiro Kawaoka (Division of Virology, Department of Microbiology and Immunology, Institute of Medical Science University of Tokyo, Japan). The viruses were stored at −80°C until use.

### 2.4. Cells

Madin-Darby canine kidney (MDCK) cells were a kind gift from Professor Hideto Fukushi (United Graduate School of Veterinary Sciences, Gifu University) and were maintained in *α*-minimal essential medium (MEM) containing 10% fetal bovine serum (FBS) and penicillin-streptomycin.

### 2.5. Virus Preparation in Cell Culture and Determination of the TCID_50_ Value

MDCK cells were cultured in T-75 culture flasks. Confluent cells were washed with phosphate-buffered saline (PBS) and then incubated with the virus (0.1 multiplicity of infection (MOI)) in 2 mL of viral growth medium (D-MEM (Dulbecco's Modified Eagle Medium) containing 0.625 *μ*g/mL of trypsin and 0.1% bovine serum albumin (BSA)) for 1 hr at 37°C. Then, 10 mL of viral growth medium was added, and the cells were incubated for 24 hrs at 37°C. The culture supernatant was harvested by centrifugation (3000 rpm at 4°C) and stored at −80°C until use. TCID_50_ (50% tissue culture infective dose) values were determined by infecting the MDCK cells with serial dilutions of the viral suspension in 96-well microtiter plates, and the TCID_50_ values were calculated using the method of Reed and Muench [35] after 6 days.

### 2.6. Mouse Adaptation and Preparation of Virus

Viruses that had been passaged 2 or 3 times in MDCK cells were further serially passaged in 6-week-old female BALB/c mice. The first passaged virus lot was obtained by intranasally inoculating a 20-*μ*L suspension of the starting virus (5.1 × 10^8^ TCID_50_ per mL) into each animal, which had been lightly anesthetized using diethyl ether. At 48 hrs postinoculation (hpi), the mice were sacrificed. Their lungs were removed, placed into PBS (2 mL for the lungs of two mice) supplemented with 0.1% BSA and penicillin-streptomycin, and homogenized using a PRO200 homogenizer (PRO Scientific Inc.; Oxford, CT, USA). The suspension was further centrifuged at 3000 rpm at 4°C, stored at −80°C, and termed MP1 (mouse passage 1). The subsequent passages were similarly performed by inoculating 40 *μ*L of the viral solution of the previous passage into each mouse. The second- and higher-passage virus lots were termed MP2, MP3, and so forth.

### 2.7. Anti-Influenza Therapeutic Efficacy in Mice

#### 2.7.1. Assessment of Survival Time

Six-week-old female BALB/c mice were lightly anesthetized with diethyl ether and inoculated with 40 *μ*L of a mixture of MP6 to MP12 (i.e., mouse-adapted viruses (8.3–53 × 10^4^ TCID_50_/mouse, resp.)) once per day for 4 days via intranasal instillation. The mice were categorized into 6 groups: (1) control (vehicle); (2) oseltamivir phosphate (0.5 mg/kg of body weight); (3) PWE (100 mg/kg); (4) PEE (100 mg/kg); (5) 3,4-diCQA (99% purity; 50 mg/kg); (6) chlorogenic acid (50 mg/kg). The compounds (in a 5% Arabic gum solution) were orally administered to the mice twice per day at intervals of more than 6 hrs for 6 days (from 0 to 5 dpi (days postinfection)). Administration began 4 hrs before viral inoculation. Mouse survival was observed once per day for 12 days after viral inoculation.

#### 2.7.2. Assessment of mRNA Expression in the Lungs

Six-week-old female BALB/c mice were lightly anesthetized with diethyl ether and inoculated with 20 *μ*L of MP14, a mouse-adapted virus (4.9 × 10^5^ TCID_50_/mouse) once per day for 2 days via intranasal instillation. The mice were categorized into three groups: (1) control (vehicle); (2) 3,4-diCQA (82% purity; 50 mg/kg); (3) oseltamivir phosphate (0.5 mg/kg). Each compound (in a 5% Arabic gum solution) was orally administered to the mice twice per day at 6-hour intervals for 8 days (from 0 to 7 dpi). Administration began 4 hrs before viral inoculation. In the mRNA expression study (*n* = 13–14 at 0 dpi), the 6 mice with the lowest body weight in each group and the remaining surviving mice in each group were killed at 4 and 7 dpi, respectively, and the mRNA expression in the lungs was determined using quantitative real-time PCR (qPCR), as described below.

Total RNA was extracted from the lung homogenate using the TriPure Isolation Reagent (Roche Diagnostics, Mannheim, Germany), according to the manufacturer's protocol. Briefly, the mice were sacrificed, and their lungs were removed, placed into a solution (1.5 mL for the lungs of one mouse) of TriPure, and homogenized using a PRO200 homogenizer. After centrifugation, the water phase of the lysate was recovered, and RNA was precipitated with the ethanol and rinsed. The purified RNA was dissolved in 50–100 *μ*L of nuclease-free water. The amount of RNA was estimated using a NanoDrop (Thermo Scientific, Wilmington, DE, USA). The cDNA from a 500-ng aliquot was synthesized using a PrimeScript RT reagent kit (Takara Bio Inc., Shiga, Japan) and oligo dT primers as RT (reverse transcription) primers, according to the manufacturer's protocol. Quantitative real-time PCR for the IAV or mouse gene was then performed. Briefly, the aliquot of the RT reaction solution was amplified using SYBR Premix Ex Taq II (Takara Bio Inc., Shiga, Japan) and a Real-Time PCR Thermal Cycler (“Thermal Cycler Dice Real Time,” Takara Bio Inc., Shiga, Japan), according to the manufacturer's protocol. H1N1-specific primers were selected based on HA (hemagglutinin) mRNA using Primer Express software (PE Applied Biosystems, Waltham, MA, USA). The sequences of the HA primer sets were 5′-CAATGTATGCTTTCGCACTGAGTA-3′ for the forward primer and 5′-GACACTTCGTGTTACACTCATGCA-3′ for the reverse primer. The sequences of the mouse TRAIL primer sets have been described elsewhere [36]. The mouse HPRT (hypoxanthine guanine phosphoribosyl transferase) primer sets were purchased from Takara Bio Inc. (Shiga, Japan). The relative quantities of H1N1 HA mRNA and mouse TRAIL mRNA were normalized to the mRNA expression of mouse HPRT (a housekeeping gene).

### 2.8. Statistics

The survival times were statistically compared using the Kaplan-Meier log-rank test and pairwise multiple comparison procedures (the Holm-Sidak method) using StatView 3.5 (SAS Institute, Cary, NC, USA). The body weights were compared using one-way analysis of variance (ANOVA). When any significance was detected by ANOVA, Dennett's nonparametric test was then applied using Ekuseru-Tokei 2006 (Social Survey Research Information Co., Ltd., Tokyo Japan). The relative mRNA expression of the target gene was compared between treatment groups using a paired *t*-test or Student's *t*-test. A *P* value of less than 0.01 (*P*< 0.01) was considered to be statistically significant.

## 3. Results

### 3.1. PWE, PEE, and 3,4-diCQA Increase the Survival Times of Mice Infected with the Influenza Virus

In preliminary experiments, MDCK culture-derived WSN/33 (H1N1, IAV) was only moderately lethal to mice, so we adapted the parent WSN/33 to mice using serial passages to enhance its virulence [37–39], as described in the Section 2. Mouse-adapted WSN/33 was able to kill more than 80% of mice within 10 days when the mice inoculated daily for 3 to 4 days. The anti-influenza activities of PWE, PEE, 3,4-diCQA, chlorogenic acid, and oseltamivir phosphate were evaluated using the mouse-adapted WSN/33 strains. 

**Figure 1:**
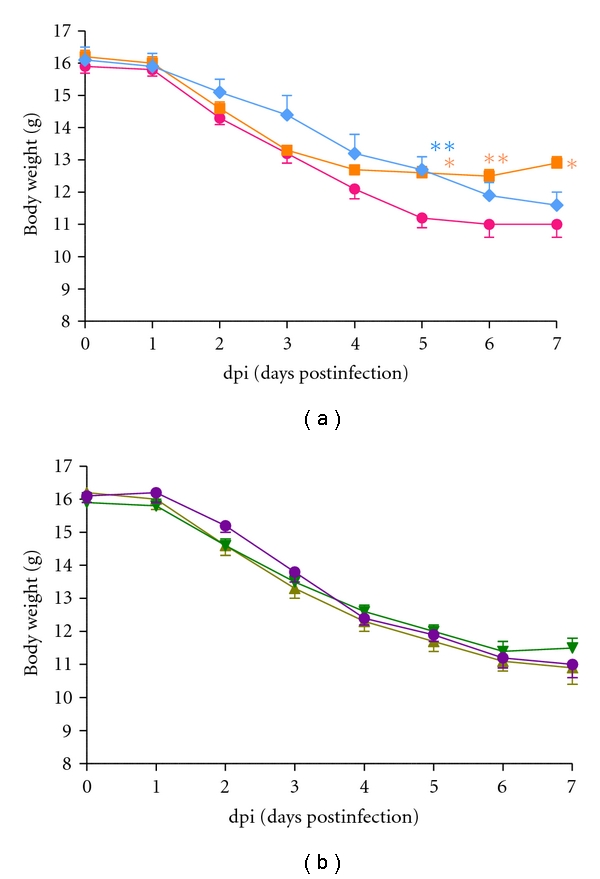
Effects of orally administrated oseltamivir phosphate, PWE, PEE, 3,4-diCQA, and chlorogenic acid on the body weight of BALB/c mice infected with the IAV. (a) control (*⚫*), oseltamivir (0.5 mg/kg; ■), 3,4-diCQA (50 mg/kg; *◆*), (b) PWE (100 mg/kg; ▲), PEE (100 mg/kg; ▾), or chlorogenic acid (50 mg/kg; *⚫*) was administrated twice daily starting at 4 hours before the first viral infection until 5 days postinfection (5 dpi). The data represent the mean ± SE for the mice that survived. *P< 0.05 and **P< 0.01 versus vehicle control.

The vehicle treatment (control) group exhibited a significant decrease in body weight from 0 to 7 dpi. Treatment with PWE, PEE, or chlorogenic acid did not affect this phenotype compared to the control. However, 3,4-CQA (at 5 dpi alone, *P*< 0.01) and oseltamivir (at 5 dpi, *P*< 0.05; at 6-7 dpi, *P*< 0.01) slightly and strongly, respectively, counteracted the body weight loss caused by the viral infection (Figures 1(a) and 1(b)).

**Figure 2:**
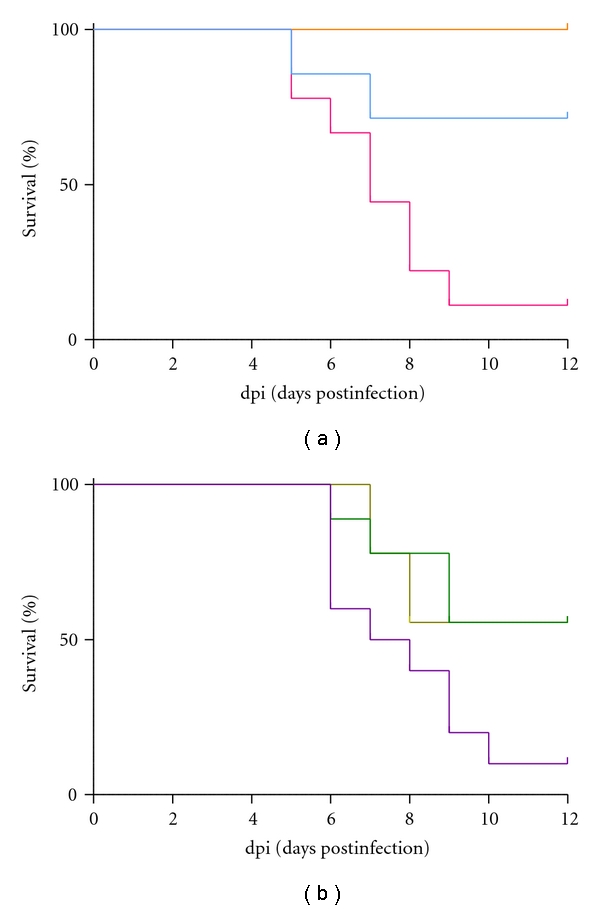
Survival curves of BALB/c mice infected with the IAV to assess the *in vivo* effects of orally administrated (a) control, oseltamivir (0.5 mg/kg), 3,4-diCQA (50 mg/kg), (b) PWE (100 mg/kg), PEE (100 mg/kg), or chlorogenic acid (50 mg/kg). The colors correspond to those used in Figures 1(a) and 1(b). The infected mice were observed for 12 days. The statistical results are shown in Table 1.

In the same experiment, 88.9% of mice in the control group died by 12 dpi, while all of the oseltamivir-treated mice survived. The survival time of the oseltamivir group was significantly longer than that of the control group (Figure 2(a), Table 1). Moreover, although chlorogenic acid had no effect, PWE, PEE, or 3,4-CQA treatment increased the survival rate (Figures 2(a) and 2(b), Table 1).

### 3.2. 3,4-diCQA Increases TRAIL Expression but Decreases HA mRNA Expression in Mouse Lungs Infected with IAV

**Figure 3:**
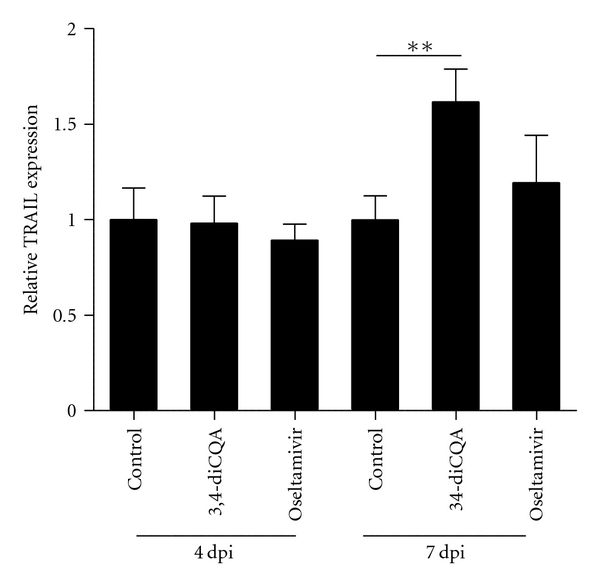
TRAIL expression in the lungs of BALB/c mice infected with the IAV. Vehicle, 3,4-diCQA (50 mg/kg), or oseltamivir (0.5 mg/kg) was administrated twice daily starting at 4 hrs before the first viral infection until 7 days post-infection (7 dpi). Total RNA was recovered at 4 dpi or 7 dpi, and relative mRNA expression was measured using real-time PCR. The quantity of mouse TRAIL mRNA expression was normalized to the mRNA expression of mouse HPRT. The quantities are shown as mean ± SE. **P< 0.01.

We examined TRAIL and HA mRNA expression in the lungs of mice infected with IAV. Treatment with 3,4-CQA or oseltamivir had no effect on TRAIL mRNA expression at 4 dpi (Figure 3). At 7 dpi, the 3,4-CQA treatment group exhibited an increase in TRAIL mRNA expression (*P*< 0.05), while the oseltamivir treatment group exhibited no change in the expression of TRAIL mRNA (Figure 3).

**Figure 4:**
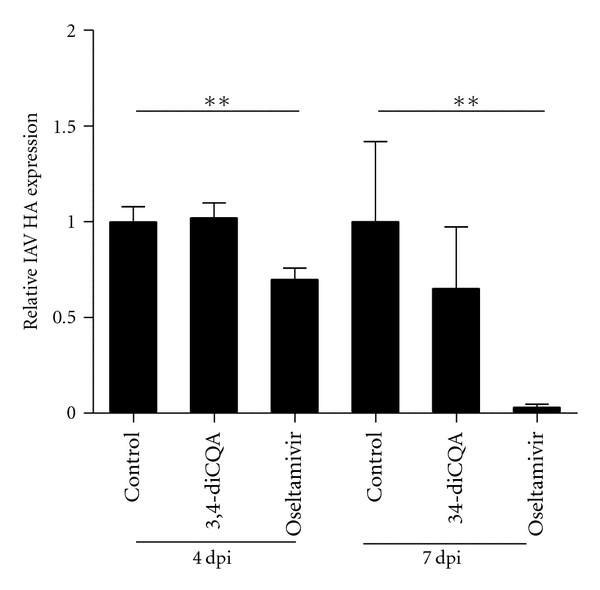
HA expression in lungs. IAV HA mRNA expression was normalized to the mRNA expression of mouse HPRT. The quantities are shown as mean ± SE. **P< 0.01 (Student's *t*-test).

In the same experiment, the oseltamivir treatment group exhibited less HA mRNA expression than the control at 4 and 7 dpi (*P*< 0.05; Figure 4). The 3,4-diCQA treatment group exhibited similar HA mRNA expression at 4 dpi and slightly less expression at 7 dpi than the control group (Figure 4). 

Overall, oseltamivir treatment decreased IAV HA mRNA expression rapidly after infection and had no effect on TRAIL mRNA expression, whereas 3,4-diCQA treatment moderately decreased IAV HA mRNA expression and increased TRAIL mRNA expression with a delay.

## 4. Discussion

First, we must point out that there are some controversies regarding the anti-influenza effects of various propolis-related substances, especially in *ex vivo* versus *in vivo* experiments. We have shown that PWE and PEE can increase the lifetimes of mice with IAV infections. In a previous report, anti-influenza activity was found for PWE [31] but not PEE (personal communication, Urushisaki T.) when using MDCK cells. Here, we speculate that, in *ex vivo *experiments, some substances (as yet unidentified) in PEE are cytotoxic to MDCK cells and may mask its anti-influenza activity. We have also shown that 3,4-diCQA can increase the lifetimes of mice with IAV infections (Table 1). 3,4-diCQA, a natural product, is an ester of two polyphenolic caffeic acids and one cyclitol (−)-quinic acid. While chlorogenic acid (a natural product, an ester of one caffeic acid and one cyclitol (−)-quinic acid) does not have anti-influenza activity *in vivo*, these two compounds have anti-influenza activity *ex vivo* [31]. This may occur due to differences in the pharmacokinetic properties of these compounds *in vivo*. 

The major chemical ingredients in PWE and PEE are listed in Table 2 [40, 41], and these show that the amount of 3,4-diCQA in PWE and PEE is 3.3–6.1% and 1.9–3.5%, respectively. It must be noted that the anti-influenza activities of PWE and PEE can only be partially explained by 3,4-diCQA. Thus, it is very likely that unknown compounds that efficiently act against the IAV are also contained in PWE and PEE.

We have also shown that 3,4-diCQA increases the mRNA expression of TRAIL in the lungs of IAV-infected mice (Figure 4). Although TRAIL is an apoptosis-inducing factor in tumor cells [42], it also induces the apoptosis of influenza virus-infected cells in infected animals via the TRAIL receptor (DR5), whose expression is induced by the virus. Ishikawa et al. [36] have shown that TRAIL mRNA expression is induced by an influenza virus infection in mouse lungs, and an anti-TRAIL monoclonal antibody (mAb) delays influenza virus clearance in mice. Moreover, Brincks et al. [43] have shown that morbidity and the influenza virus titer are increased in TRAIL-knockout (TRAIL^−/−^) mice. Therefore, TRAIL is an important anti-influenza factor with viral clearance activity.

3,4-diCQA did not affect TRAIL expression at 4 dpi (Figure 3). However, the infected animals were protected even at early stages of the infectious period without increased TRAIL expression because 3,4-diCQA increases the viability of infected cells, as suggested by previous *ex vivo* experiments [31]. It must be noted that MDCK cells do not express the TRAIL receptor [44]. 3,4-diCQA induced TRAIL mRNA at 7 dpi relative to the control group (Figure 3). During the middle stage of infection, TRAIL expression increased, thereby increasing the lifetimes of the mice.

It has also been reported that the induction of TRAIL mRNA depends on NF-*κ*B [45]. However, at the current stage, it is unclear whether the enhancement of TRAIL mRNA expression by 3,4-diCQA is due to NF-*κ*B activation.

We confirmed that Brazilian green propolis extracts (PWE and PEE) have anti-influenza activities. Here, we also hypothesized that their mode of action at least partially includes two mechanisms: an unknown cytoprotective mechanism [31] and the enhancement of viral clearance via TRAIL overexpression. We hypothesize that both are induced by 3,4-diCQA and/or unknown active constituents of Brazilian green propolis.

An important point regarding our hypothesis concerning the anti-influenza effects of propolis or its constituent, 3,4-diCQA, is that it might trigger or enhance the self-defense machineries of the host. Although viruses can easily become resistant to anti-influenza drugs, such as oseltamivir, that directly target viral proteins [4, 5], it would be more difficult for viruses to become resistant to antiviral agents whose target molecules are host molecules or machineries. Therefore, health supplements such as propolis may be useful as an alternative strategy for protection against influenza virus infections.

Many traditional Chinese medicinal herbs appear to have potential anti-influenza effects [46], but their mechanisms are not well understood. Our findings show that PWE and PEE and their constituent, 3,4-diCQA, may be useful as a potent lead compound for anti-influenza medicine. This may promote research into anti-influenza medicine developed from traditional substances.

## Figures and Tables

**Table 1:** Survival of mice infected with IAV after the oral administration of various substances.

Treatment	*n*	Dose	Duration (dpi)	Survival time (dpi, mean ± SE)	Statistical significance
5% Arabic gum	9		0–5	7.4 ± 0.7	
oseltamivir	8	0.5 mg twice/kg/day	0–5	>12.0^ #^	**
PWE	9	100 mg twice/kg/day	0–5	10.0 ± 0.9	*
PEE	9	100 mg twice/kg/day	0–5	10.1 ± 0.9	*
3,4-diCQA	7	50 mg twice/kg/day	0–5	10.3 ± 1.5	*
Chlorogenic acid	9	50 mg twice/kg/day	0–5	8.1 ± 0.8	N.S.

^#^standard error not determined; *P < 0.05; **P < 0.01; N.S. not significant.

**Table 2:** Concentrations and molecular weights of the constituents of Brazilian green propolis [40, 41].

Components	Content (w/w%) in PWE	Content (w/w%) in PEE	Molecular weight (g/mol)
Chlorogenic acid	2.7–3.6	0.6–0.8	354.3
Caffeic acid	0.2	0.1–0.6	180.2
3,5-dicaffeoylquinic acid	4.3–4.9	2.4–2.7	516.5
3,4-dicaffeoylquinic acid	3.3–6.1	1.9–3.5	516.5
4,5-dicaffeoylquinic acid	—^#^	—^#^	516.5
3,4,5-tricaffeoylquinic acid	0.2	0.6	678.6
Artepillin C	0.2–0.6	11.4–14.0	300.4
Baccharin	0.0	6.8	562.6
Drupanin	0.1	1.8	232.3
Isosakuranetin	—^#^	1.2	286.3
*p*-coumaric acid	3.7	2.3–2.5	164.2
Ferulic acid	0.1	0.0–0.1	194.2
Quinic acid	—^#^	—^#^	192.2

^#^Data not available.

## References

[1] Hayden FG, Osterhaus ADME, Treanor JJ (1997). Efficacy and safety of the neuraminidase inhibitor zanamivir in the treatment of influenza virus infection. *The New England Journal of Medicine*.

[2] Nicholson KG, Aoki FY, Osterhaus AD (2000). Efficacy and safety of Oseltamivir in treatment of acute influenza: a randomised controlled trial. Neuraminidase Inhibitor Flu Treatment Investigator Group. *The Lancet*.

[3] Treanor JJ, Hayden FG, Vrooman PS (2000). Efficacy and safety of the oral neuraminidase inhibitor oseltamivir in treating acute influenza: a randomized controlled trial. *Journal of the American Medical Association*.

[4] Ciblak MA, Hasoksuz M, Escuret V (2009). Surveillance and oseltamivir resistance of human influenza A virus in Turkey during the 2007-2008 season. *Journal of Medical Virology*.

[5] Tamura D, Mitamura K, Yamazaki M (2009). Oseltamivir-resistant influenza A viruses circulating in Japan. *Journal of Clinical Microbiology*.

[6] Aggarwal S, Bradel-Tretheway B, Takimoto T, Dewhurst S, Kim B (2010). Biochemical characterization of enzyme fidelity of influenza a virus RNA polymerase complex. *PLoS ONE*.

[7] Nelson MI, Viboud C, Simonsen L (2008). Multiple reassortment events in the evolutionary history of H1N1 influenza A virus since 1918. *PLoS Pathogens*.

[8] Bankova V, Marcucci MC, Simova S, Nikolova N, Kujumgiev A, Popov S (1996). Antibacterial diterpenic acids from Brazilian propolis. *Zeitschrift fur Naturforschung*.

[9] Drago L, Mombelli B, De Vecchi E, Fassina MC, Tocalli L, Gismondo MR (2000). In vitro antimicrobial activity of propolis dry extract. *Journal of Chemotherapy*.

[10] Abd El Hady FK, Hegazi AG (2002). Egyptian propolis: 2. Chemical composition, antiviral and antimicrobial activities of East Nile Delta propolis. *Zeitschrift fur Naturforschung*.

[11] Nakajima Y, Shimazawa M, Mishima S, Hara H (2007). Water extract of propolis and its main constituents, caffeoylquinic acid derivatives, exert neuroprotective effects via antioxidant actions. *Life Sciences*.

[12] Mirzoeva OK, Calder PC (1996). The effect of propolis and its components on eicosanoid production during the inflammatory response. *Prostaglandins Leukotrienes and Essential Fatty Acids*.

[13] Chen CN, Weng MS, Wu CL, Lin JK (2004). Comparison of radical scavenging activity, cytotoxic effects and
apoptosis induction in human melanoma cells by taiwanese propolis
from different sources. *Evidence-Based Complementary and Alternative Medicine*.

[14] Kujumgiev A, Tsvetkova I, Serkedjieva Y, Bankova V, Christov R, Popov S (1999). Antibacterial, antifungal and antiviral activity of propolis of different geographic origin. *Journal of Ethnopharmacology*.

[15] Markham KR, Mitchell KA, Wilkins AL, Daldy JA, Lu Y (1996). HPLC and GC-MS identification of the major organic constituents in New Zealand propolis. *Phytochemistry*.

[16] Mohamed RF, Owayss AA (2005). An inhibitory activity of propolis extract against broadbean
mottle bromovirus (BBMV). *International Journal of Virology*.

[17] Amoros M, Simoes CMO, Girre L, Sauvager F, Cormier M (1992). Synergistic effect of flavones and flavonols against herpes simplex virus type 1 in cell culture. Comparison with the antiviral activity of propolis. *Journal of Natural Products*.

[18] Amoros M, Lurton E, Boustie J, Girre L, Sauvager F, Cormier M (1994). Comparison of the anti-herpes simplex virus activities of propolis and 3- methyl-but-2-enyl caffeate. *Journal of Natural Products*.

[19] Huleihel M, Isanu V (2002). Anti-herpes simplex virus effect of an aqueous extract of propolis. *Israel Medical Association Journal*.

[20] Nolkemper S, Reichling J, Sensch KH, Schnitzler P (2010). Mechanism of herpes simplex virus type 2 suppression by propolis extracts. *Phytomedicine*.

[21] Schnitzler P, Neuner A, Nolkemper S (2010). Antiviral activity and mode of action of propolis extracts and selected compounds. *Phytotherapy Research*.

[22] Búfalo MC, Figueiredo AS, De Sousa JPB, Candeias JMG, Bastos JK, Sforcin JM (2009). Anti-poliovirus activity of Baccharis dracunculifolia and propolis by cell viability determination and real-time PCR. *Journal of Applied Microbiology*.

[23] Fahmy FG, Omar MO Effect of propolis extract on certain potato viruses.

[24] Ito J, Chang FR, Wang HK (2001). Anti-AIDS agents. 48. Anti-HIV activity of moronic acid derivatives and the new melliferone-related triterpenoid isolated from Brazilian propolis. *Journal of Natural Products*.

[25] Maximova-Todorova V, Manolova N, Gegova G (1985). Antiviral effects of some fractions isolated from propolis. *Acta Microbiologica Bulgarica*.

[26] Amoros M, Sauvager F, Girre L, Cormier M (1992). In vitro antiviral activity of propolis. *Apidologie*.

[27] Shevchenko LF

[28] Esanu V, Prahoveanu E, Crisan J, Cioca A (1981). The effect of an aqueous propolis extract, of rutin and of a rutin-quercetin mixture on experimental influenza virus infection in mice. *Revue Roumaine de Medecine-Serie de Virologie*.

[29] Serkedjieva J, Manolova N, Bankova V (1992). Anti-influenza virus effect of some propolis constituents and their analogues (esters of substituted cinnamic acids). *Journal of Natural Products*.

[30] Shimizu T, Hino A, Tsutsumi A, Yong KP, Watanabe W, Kurokawa M (2008). Anti-influenza virus activity of propolis in vitro and its efficacy against influenza infection in mice. *Antiviral Chemistry and Chemotherapy*.

[31] Urushisaki T, Takemura T, Tazawa S (2011). Caffeoylquinic acids are major constituents with potent anti-influenza effects in Brazilian green propolis water extract. *Evidence-Based Complementary and Alternative Medicine*.

[32] Ewelina A, Zenon PC, Joanna B, Anna M, Andrej P, Wojciech K (2011). Ethanolic extract of propolis augments TRAIL-induced apoptotic death in prostate cancer cells. *Evidence-Based Complementary and Alternative Medicine*.

[33] Kumazawa S, Yoneda M, Shibata I, Kanaeda J, Hamasaka T, Nakayama T (2003). Direct evidence for the plant origin of Brazilian propolis by the observation of honeybee behavior and phytochemical analysis. *Chemical and Pharmaceutical Bulletin*.

[34] Neumann G, Watanabe T, Ito H (1999). Generation of influenza A viruses entirely from cloned cDNAs. *Proceedings of the National Academy of Sciences of the United States of America*.

[35] Reed LJ, Muench H (1938). A simple method of estimating fifty per cent endpoints. *American Journal of Epidemiology*.

[36] Ishikawa E, Nakazawa M, Yoshinari M, Minami M (2005). Role of tumor necrosis factor-related apoptosis-inducing ligand in immune response to influenza virus infection in mice. *Journal of Virology*.

[37] Hirst GK (1947). Studies on the mechanism of adaptation of influenza virus to mice. *The Journal of Experimental Medicine*.

[38] Raut S, Hurd J, Blandford G, Heath RB, Cureton RJ (1975). The pathogenesis of infections of the mouse caused by virulent and avirulent variants of an influenza virus. *Journal of Medical Microbiology*.

[39] Wyde PR, Couch RB, Mackler BF (1977). Effects of low and high passage influenza virus infection in normal and nude mice. *Infection and Immunity*.

[40] Mishima S, Yoshida C, Akino S, Sakamoto T (2005). Antihypertensive effects of Brazilian propolis: identification of caffeoylquinic acids as constituents involved in the hypotension in spontaneously hypertensive rats. *Biological and Pharmaceutical Bulletin*.

[41] Izuta H, Narahara Y, Shimazawa M, Mishima S, Kondo SI, Hara H (2009). 1,1-diphenyl-2-picrylhydrazyl radical scavenging activity of bee products and their constituents determined by ESR. *Biological and Pharmaceutical Bulletin*.

[42] Bonavida B, Ng CP, Jazirehi A, Schiller G, Mizutani Y (1999). Selectivity of TRAIL-mediated apoptosis of cancer cells and synergy with drugs: the trail to non-toxic cancer therapeutics (review). *International Journal of Oncology*.

[43] Brincks EL, Katewa A, Kucaba TA, Griffith TS, Legge KL (2008). CD8 T cells utilize TRAIL to control influenza virus infection. *Journal of Immunology*.

[44] Elders RC, Baines SJ, Catchpole B (2009). Susceptibility of the C2 canine mastocytoma cell line to the effects of tumor necrosis factor-related apoptosis-inducing ligand (TRAIL). *Veterinary Immunology and Immunopathology*.

[45] Wurzer WJ, Ehrhardt C, Pleschka S (2004). NF-*κ*B-dependent induction of tumor necrosis factor-related apoptosis-inducing ligand (TRAIL) and Fas/FasL is crucial for efficient influenza virus propagation. *Journal of Biological Chemistry*.

[46] Chen XY, Wu TX, Liu GJ (2007). Chinese medicinal herbs for influenza. *Cochrane Database of Systematic Reviews*.

